# Leveraging Artificial Intelligence to Predict Potential TB Hotspots at the Community Level in Bangui, Republic of Central Africa

**DOI:** 10.3390/tropicalmed10040093

**Published:** 2025-04-03

**Authors:** Kobto G. Koura, Sumbul Hashmi, Sonia Menon, Hervé G. Gando, Aziz K. Yamodo, Anne-Laure Budts, Vincent Meurrens, Saint-Cyr S. Koyato Lapelou, Olivia B. Mbitikon, Matthys Potgieter, Caroline Van Cauwelaert

**Affiliations:** 1 International Union Against Tuberculosis and Lung Disease, 75001 Paris, France; sonia.menon.consultant@theunion.org (S.M.); herve.gando.consultant@theunion.org (H.G.G.); aziz.yamodo.consultant@theunion.org (A.K.Y.); olivia.mbitikon.consultant@theunion.org (O.B.M.); 2 UMR261 MERIT, Université Paris Cité, IRD, 75006 Paris, France; 3 EPCON, 2050 Antwerpen, Belgium; sumbul@epcon.ai (S.H.); anne-laure@epcon.ai (A.-L.B.); vincent@epcon.ai (V.M.); thys@epcon.ai (M.P.); 4 Epitech Research, 1160 Auderghem, Belgium; 5 National Tuberculosis Programme, Bangui P.O. Box 883, Central African Republic; 6 National Health Information System, Bangui P.O. Box 883, Central African Republic; sylvanuskoyato@gmail.com

**Keywords:** machine learning, surveillance enhancement, public health, low-income countries

## Abstract

Tuberculosis (TB) is a global health challenge, particularly in the Central African Republic (CAR), which is classified as a high TB burden country. In the CAR, factors like poverty, limited healthcare access, high HIV prevalence, malnutrition, inadequate sanitation, low measles vaccination coverage, and conflict-driven crowded living conditions elevate TB risk. Improved AI-driven surveillance is hypothesized to address under-reporting and underdiagnosis. Therefore, we created an epidemiological digital representation of TB in Bangui by employing passive data collection, spatial analysis using a 100 × 100 m grid, and mapping TB treatment services. Our approach included estimating undiagnosed TB cases through the integration of TB incidence, notification rates, and diagnostic data. High-resolution predictions are achieved by subdividing the area into smaller units while considering influencing variables within the Bayesian model. By designating moderate and high-risk hotspots, the model highlighted the potential for precise resource allocation in TB control. The strength of our model lies in its adaptability to overcome challenges, although this may have been to the detriment of precision in some areas. Research is envisioned to evaluate the model’s accuracy, and future research should consider exploring the integration of multidrug-resistant TB within the model.

## 1. Introduction

Tuberculosis (TB) continues to pose a significant global public health challenge, particularly in resource-constrained settings like the Central African Republic (CAR), which reported an estimated TB incidence rate of 540 per 100,000 people in 2020 [1], designating it as a high TB burden country. The situation is compounded by endemic poverty [2], limited access to health care, and one of the highest HIV prevalence rates in Central and Western Africa [3], estimated at 3.4% [2.5–5.2] among adults aged 15–49 in 2022 [4].

In addition to these systemic challenges, TB risk is further elevated by malnutrition [5], a lack of clean water and sanitation [6], and overcrowded living conditions exacerbated by ongoing conflict [7]. These factors collectively create a setting highly conducive to TB transmission and highlight the pressing need for comprehensive prevention strategies supported by reliable surveillance systems.

Bangui, the capital city, exemplifies these challenges in an urban context. With a 2023 population of 958,000 and a rapid 2.68% annual growth rate [8], the increasing population density in Bangui adds urgency to TB control efforts, as similar urban dynamics have been associated with elevated TB risk in countries like Brazil [9]. However, the current CAR’s TB surveillance system remains reliant on traditional approaches, which are insufficient for capturing the true burden of disease and result in significant under-reporting. Therefore, strengthening surveillance systems is crucial for enabling timely detection and intervention.

Recent advances in artificial intelligence (AI) offer promising tools to address these challenges. AI has shown its potential in disease modeling, hotspot detection, and predictive analytics, particularly during the COVID-19 pandemic [10]. It can enhance surveillance systems by integrating diverse datasets, analyzing patterns, and identifying areas of elevated disease risk [11]. Applied to TB, AI may enable the identification of real-time hotspots, support targeted interventions, and reveal previously undetected transmission dynamics—especially when high-resolution facility and contextual data are incorporated.

The CAR National Tuberculosis Program (NTP) reported a gap of 15,784 missing TB cases in 2021, underscoring the need for improved detection. While notified cases are increasing, the gap between estimated and reported cases remains significant [1], largely due to underdiagnosis and under-reporting. In response, the 2024–2028 strategic plan prioritizes expanding service coverage in districts with lower case notifications (from 45% to 90%), improving detection through molecular diagnostics and radiography, targeting vulnerable populations, and increasing multidrug-resistant TB (MDR-TB) notification rates from 19% in 2021 to 90% at the end of 2028.

To support these goals, we conducted a proof-of-concept study in Bangui in collaboration with the Union, EPCON (a company specializing in AI for public health), and the NTP. Our objective was to predict potential TB hotspots at the community level and identify underserved areas to optimize case detection and treatment. Through advanced modeling and geospatial visualization, we aimed to enhance the national TB control strategy by uncovering hidden patterns in disease transmission and informing targeted public health interventions.

## 2. Materials and Methods

### 2.1. Setting

**Figure 1:**
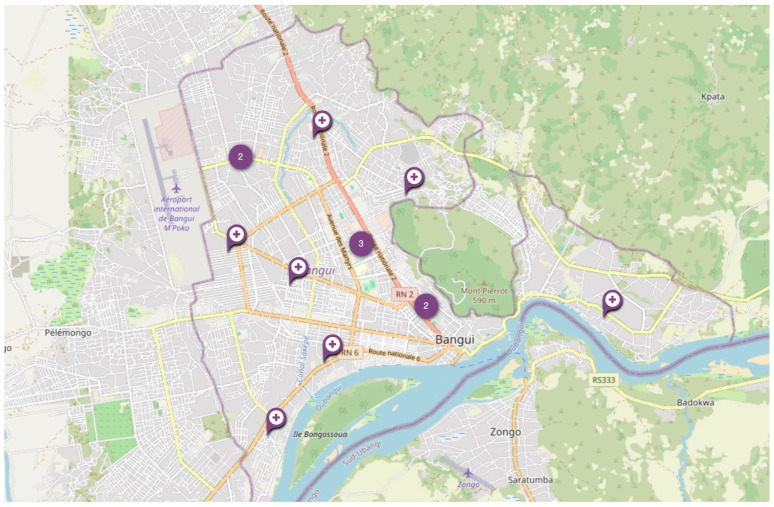
Locations of TB clinics in Bangui, CAR.

Our research included 14 TB clinics in Bangui (Figure 1). Although, due to the size of the city (67 km^2^), most inhabitants should have access, it is noteworthy that the North and the extreme East have fewer TB clinics.

**Figure 2:**
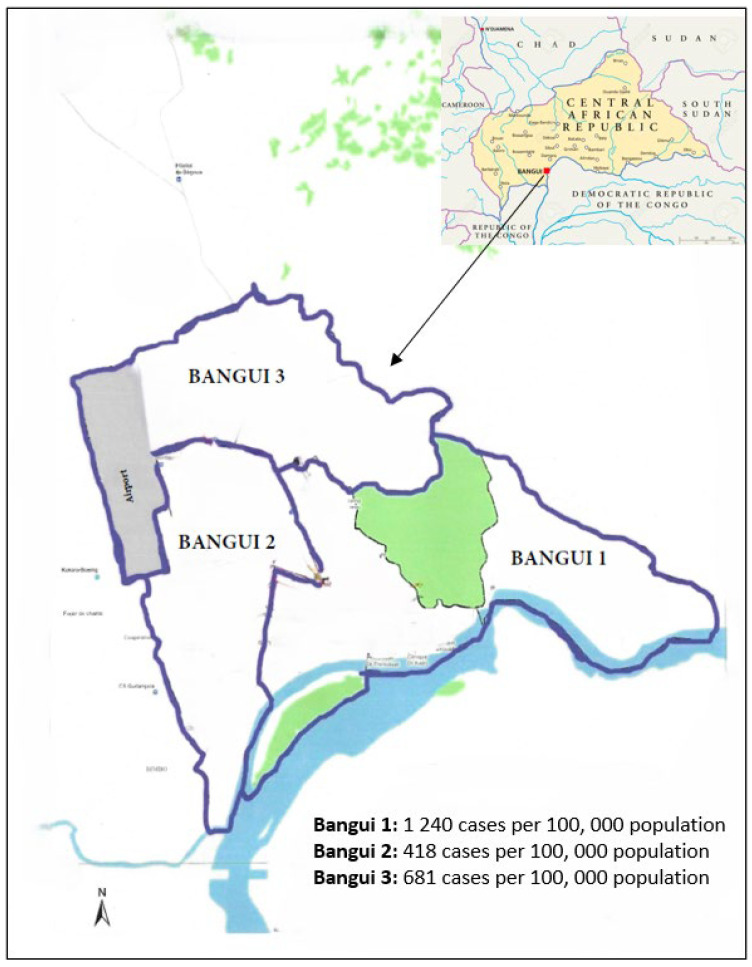
Notification rate of new cases and relapses for the three districts of Bangui, 2021.

The analysis of notification rates indicates significant disparities, with 1240, 418, and 681 cases per 100,000 inhabitants observed in Bangui 1, Bangui 2, and Bangui 3 districts, respectively (Figure 2).

### 2.2. Data Collection

We employed a comprehensive methodology to develop an epidemiological digital representation of the TB situation in Bangui city. Our approach was initiated with data collection and preparation by means of a thorough mapping of population settlements within the city, providing insights into the distribution of residents across the geographic region. Simultaneously, we introduced a fine-grained grid with a resolution of 100 × 100 m, overlaying it onto the study area, which in turn enabled us to capture intricate spatial variations.

We intended to obtain an estimate of undiagnosed TB potentially present in Bangui at the highest resolution. We identified and mapped all TB clinics operating within the study area. Data on number of TB clients registered at each clinic was available only as aggregated numbers. Total population of the city was mapped on a grid of 100 × 100 m tiles. Since no other disaggregated information was available, we leveraged a few assumptions. First, we defined clinic catchment areas (CCA) as the geographic zones from which the majority of patients are likely to seek care at a given clinic based on proximity. Operationally, we used a 20 min travel time threshold to delineate each CCA, assuming that individuals living within this radius are more likely to attend the corresponding facility for TB services. This choice ensured that we considered practical accessibility to TB treatment services within the community. The CCA also served to provide a view of the population that lived too far from any TB clinic. In order to design these CCA, we mapped the locations of the 14 TB clinics on the tiles map for Bangui. Then, using the openstreet map, we calculated travel times to each facility from the centroid of each tile. That gave us 14 maps of travel time (one for each TB clinic) at the tile level. The value associated with each tile then becomes the travel time. Thereafter, we filtered the 14 tile-level maps of travel time to the corresponding facilities by excluding tiles beyond a 20 min travel time. This resulted in 14 catchment areas at the tile level where people can reach each TB clinic within 20 min.

Second, we assumed that the difference between TB incidence rate (national-level estimates from WHO) and TB notification rate (as available from clinic registers) could serve as an indicator of undiagnosed or unnotified TB cases. With the incidence rate assumed to be uniform across the city, the clinics that notified the most cases were believed to have relatively fewer undiagnosed or unnotified TB cases remaining within their catchment area. Using this approach, we calculated the rate of undiagnosed or unnotified TB cases for each CCA. Subsequently, this rate was distributed across all tiles in proportion to their population. To extrapolate our findings across the entire city, we developed a predictive model grounded in Bayesian methodology. This choice was driven by its effectiveness in managing uncertainty in complex environments. In our case, the complexity encompassed TB clinics and socio-economic data, multiple variables with potentially nonlinear relationships, significant natural variation, and missing values. The output of this first model was the predicted rate of undiagnosed or unnotified TB cases at the resolution of 100 m × 100 m. As this approach involved significant assumptions, we opted for an alternate strategy for using notification data.

Later, in collaboration with the TB clinic staff, we identified the aggregated number of individuals with presumptive TB who underwent testing and were diagnosed with bacteriologically confirmed TB in each neighborhood (quartier) during 2021 and 2022 using the client residential addresses from TB clinic registries. We calculated ‘TB positivity rate’, defined as the proportion of TB positives among the individuals tested in each neighborhood. These diagnostic data were then contextualized within the neighborhoods (quartier) and weighted based on population density, reinforcing the reliability of our analyses.

The existence of significant disparities in population size, with variations spanning from small neighborhoods with just a few hundred residents to larger ones with as many as 20,000 inhabitants, could potentially hinder our ability to detect subtle variations in potential TB risk. To address this challenge, there was need for a methodological approach aimed at elevating the prediction resolution to the sub-neighborhood level. This was achieved by dividing the city’s population settlements into smaller units, each 100 m × 100 m in size. By breaking down the area this way, TB positivity rates for each neighborhood were distributed across these smaller tiles based on the population of each tile. Tiles with more people and higher crowding were attributed to higher TB positivity rates, assuming that these areas have a higher risk of TB transmission. Importantly, the average TB positivity rate across all tiles in a neighborhood consistently preserved the original rate.

### 2.3. Data Analysis

#### 2.3.1. Integration of Relevant Variables

The prediction of the TB burden was based on variables with established influence on its occurrence to reflect both socio-environmental and health risk factors for TB. Socioeconomic–environmental factors included total population density [12], access to drinkable water [13], sanitation access [13], altitude [14], and male and female literacy rates [15]. As health risk factors, clinical variables such as children under 5 years [16], underweight prevalence in children under 5 years [17], and HIV prevalence [18] were chosen. Proximity to main roads and nighttime light levels, used as proxies for accessibility to TB clinics, were also included in the analysis. Additionally, child mortality rate (under 5 years), DPT [1,2,3] vaccination coverage (12–23 months), and measles vaccination rates (12–23 months) were considered as proxies for access to care and health-seeking behavior.

By considering these variables, we aimed to construct a robust predictive model that captured the multifaceted factors contributing to the TB burden. The Pearson coefficient was computed for each variable with both observed and predicted TB positivity. Table 1 describes the variables used, their definition, the source, the resolution, and the year of publication.

#### 2.3.2. Incorporation of Program Data

First, notification data were employed at the neighborhood level to calculate the rate of positive tests (diagnosed positive among the persons who were tested divided by the number of persons who were tested, and the result was multiplied by 100) within this specific level of aggregation. Following this calculation, a weighting factor was applied to redistribute the TB positivity rates to each tile based on its population density. To identify hotspots, tiles were categorized as high, moderate, or low based on TB positivity rates to identify areas of particular concern (high-risk hotspots) and areas with lesser burden (moderate- and low-risk areas).

#### 2.3.3. Application of Bayesian Model and Prediction Analysis

The Bayesian model utilized facility-level notification data to create a detailed map of TB positivity rates at a 100-square-meter resolution. With the map of the TB positivity rate based on facility-level notification data at a resolution of 100 square meters across Bangui, albeit with some missing values, we then queried the Bayesian model for predicted TB positivity rate across the city, including areas where observations were not available, using known local contextual data at 100 m resolution. The outputs corresponded to a larger geographical area, which also included population settlements in the peri-urban areas around Bangui city. The prediction at this resolution revealed a heterogeneous distribution of TB positivity across the city. Overlaying this information with locations of each TB clinic helped to identify the potential TB risk in the catchment areas of each facility, which can be targeted for further interventions.

#### 2.3.4. Model Training

The model training process involved data cleaning and normalization to ensure the input data were suitable for model training.

Most of the variables were available as rates or proportions; they were used as such. Some variables with absolute values were scaled to rates to make them comparable across different units. Missing values were imputed using the nearest neighbor algorithm. We employed a Full Bayes approach to train our model, which considered the joint distribution of all covariates, thereby capturing dependencies between them. This approach is particularly advantageous as it provides a probabilistic representation of the strength of relationships among variables, which is useful when prior knowledge of these relationships is limited or unavailable. This method aligns with the approaches discussed by Koller and Friedman (2009) [29].

To determine the relationships between variables, we utilized chance-adjusted mutual information between covariates to identify which variables are related. Conditional probabilities between each set of child states and the joint states of their parents were then calculated, with these relationships stored in conditional probability tables (CPTs). This unsupervised method allows for the accommodation of complex datasets without relying on the assumption of conditional independence between covariates. The role of entropy in determining variable relationships is detailed by MacKay (2003) [30].

We based the selection of drivers and determinants of TB burden on literature searches and known disease drivers. Variables that exhibited weak or no identifiable relationship to TB burden, as determined by Shannon’s Entropy, were either excluded from the model or weighted less heavily than those with a strong relationship to TB burden. The variables used in this study are already described in the manuscript.

We created conditional probability tables to define the relationships between predictors using the proprietary Python-based EPCON epi-control platform (Python 3.6.7 and EPCON API v1.15.1). Both the independent and dependent variables (TB risk) were discretized using percentile bucketing, with each percentile range treated as a separate class for the model to predict. The model’s output provided a probability distribution across these percentiles, from which 95% credible intervals were derived. The midpoint (50th percentile) of the estimated probability distribution was used as the predicted value. The Full Bayes approach we utilized is consistent with the methodologies described by Jordan (2003) [31].

The Bayes Theorem formula is

p(X = x | Y = y) = [p(X = x) × p(Y = y|X = x)]/p(Y = y)

The expanded form of the Bayes Theorem formula is

p(X = x|Y = y) = [p(x₁, x₂, …, xₙ|Y = y) × p(Y = y)]/Σₓ’ [p(x’₁, x’₂, …, x’ₙ|Y = y) × p(Y = y)]

where the following apply:

- p(X = x|Y = y) is the posterior probability;
- p(X = x, Y = y) is the joint probability;
- p(Y = y) is the marginal probability of Y;
- p(X = x) is the prior probability of X;
- p(Y = y|X = x) is the likelihood.

This expanded form is essential for Bayesian inference when dealing with multiple covariates, as outlined by Murphy (2012) [32].

The application of the above formulas to obtain TB risk predictions is as follows:

- Defining the Prior p(X = x): Facility-level TB notification data at a 100 m × 100 m resolution were used to determine the probability that each tile’s TB rate (X) falls within each specific percentile range. This provided the baseline TB risk for each tile without incorporating additional contextual data.
- Establishing the Likelihood p(Y = y|X = x): The likelihood specifies how probable it is to observe a particular covariate pattern (Y = y), given that TB status (or rate range) is x. Observed notification data were used to determine how socio-environmental factors correlate with TB notification rates. These probabilities are encoded in Conditional Probability Tables (CPTs). For each TB notification rate interpercentile range, the CPT indicates the likelihood of each combined set of covariate states.
- Combining Prior and Likelihood: For each 100 m × 100 m tile, the software retrieves its covariate profile y. Using the CPT, it obtains p(Y = y|X = x), reflecting the probability of those covariate ranges if TB state x is observed. The product p(X = x) × p(Y = y|X = x) gives an unnormalized posterior, a measure of how likely it is that a tile’s TB notification rate falls in a particular percentile range x, given the observed covariates.
- Normalizing to Obtain the Posterior: To ensure probabilities sum to 1, the algorithm divides by the marginal probability p(Y = y), which is the sum of all prior-likelihood products over possible TB states. After normalization, the posterior p(X = x|Y = y) denotes the updated probability of each TB rate range, having accounted for both local covariates and baseline TB rates.
- Inference at High Resolution: This procedure is repeated for each tile in the study area. The posterior distribution is calculated by the probability assigned to each TB notification interpercentile range. The midpoint (50th percentile) of the posterior distribution is used as the predicted TB positivity rate, with 95% credible intervals reflecting uncertainty.
- Calculating Predicted TB Burden: Once the posterior (i.e., the TB positivity rate) is determined for each tile, multiplying that rate by the tile’s population yields the absolute TB burden. Summing the tile-level burdens provides an estimate of total TB burden across the city.
- Software Implementation: The trained model is queried at high spatial resolution using proprietary Python-based inference software. Each tile’s covariates are fed into the CPTs, and the resulting posterior distribution informs local TB burden estimates.

### 2.4. Model Evaluation

In this study, we prioritized risk stratification over strict classification, as our primary goal is to identify high-risk areas for TB transmission rather than to classify individual cases. Given the nature of TB surveillance, public health decision making relies more on understanding spatial risk patterns than on binary classification performance. The chosen evaluation approach, which categorizes areas into risk levels based on model outputs, is aligned with epidemiological principles and facilitates actionable insights for TB control programs.

While classification metrics such as accuracy, recall, and F1-score were useful in traditional disease classification tasks, they were less suited for geospatial risk prediction models that operate on a continuum rather than discrete class labels. Instead, our model’s performance was assessed by comparing predicted TB risk estimates with known case distributions and ground-truth epidemiological data. This ensures the practical relevance of the model’s predictions for targeted intervention planning in TB-endemic settings.

### 2.5. Ethics

The study was conducted under programmatic conditions, relying on aggregated instead of personal data. Formal approval from ethic committee of the country was not necessary for this purpose. The data provided by NTP were used with the consent of program manager.

## 3. Results

### 3.1. TB Positivity Rates

**Figure 3:**
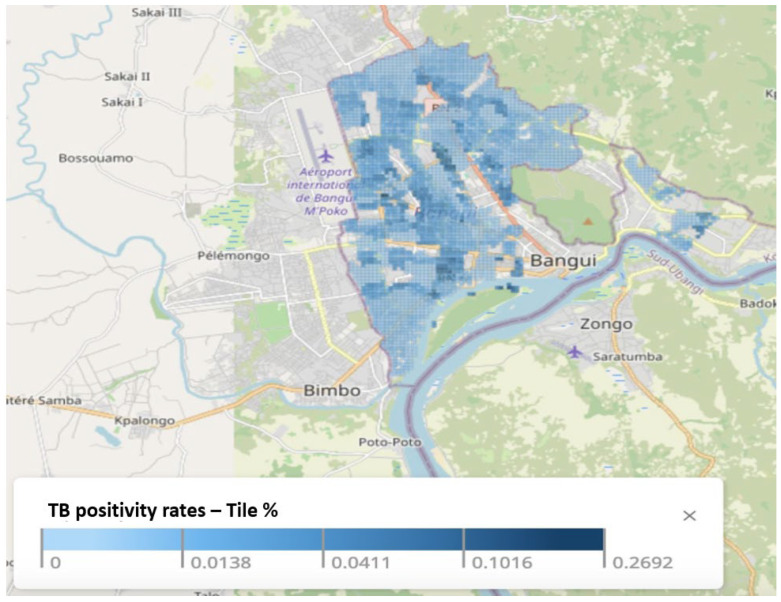
Distribution of TB positivity rates in 100 × 100 m areas in Bangui.

Our research yielded a detailed neighborhood-level notification data map that visualizes TB notifications at a high spatial resolution of 100 square meters (Figure 3). This map also provides an in-depth depiction of TB-positive notifications across the city, which enables a precise examination of TB notification patterns at the quartier level.

### 3.2. Predicted TB Positivity Rate

**Figure 4:**
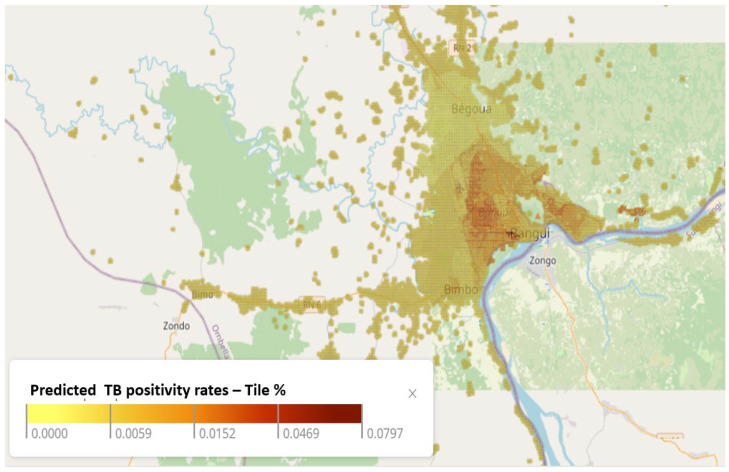
Predicted TB positivity rates in 100 × 100 m areas in Bangui.

The Bayesian model produced a detailed map with predicted TB positivity rates at a high spatial resolution of 100 square meters (Figure 4). The output was the predicted TB positivity rate across Bangui, including remote settlements, which did not fall in any of the catchment areas at a resolution of 100 m. The model allowed the identification of localities with high unmet needs for TB diagnosis and treatment services. The overall region could be categorized into three groups: high, moderate, and low rates of TB positivity. These categories were defined as follows: low risk, representing 5 or fewer TB positives per 1000 tested; medium risk, covering rates ranging from 5 to 10 TB positives per 1000 tested; and high risk, signifying rates exceeding 10 TB positives per 1000 tested. Of the 12 facilities that provide treatment services, two, seven, and three were classified to be located in high-, medium-, and low-risk regions, respectively.

### 3.3. Comparison of Notified and Predicted TB Risk in the Vicinity of TB Clinics

Seven TB clinics were classified as being located in areas with predicted profiles different from those terminated based on notification data (Table 2).

The Pearson correlation coefficients computed for predicted TB and notified TB positivity revealed notable trends, which were comparable. Among all the variables assessed, we observed moderately robust positive linear relationships between notified TB positivity rates, population density, and night-time lights, suggesting that notified TB positivity rates tended to increase as the population density and night-time light intensity (a proxy for economic activity/development) increased. In contrast, weaker positive correlations were observed with DPT 1 and 3 vaccinations, the distance to build settlements, and measles vaccination. Conversely, a weak negative linear relationship was observed for predicted TB positivity (Table 3) and notified TB positivity rates (Table 4) and the HIV incidence rate, children underweight, stunted growth, distance to major roads, and elevation, suggesting that the model predicted low TB positivity in areas where values for these variables were higher.

## 4. Discussion

The resulting Bayesian model facilitated the estimation of potential TB hotspots across the urban area of Bangui, offering a spatial assessment of the disease distribution. Notably, our findings enabled us to fine-tune the NTP map that had stratified Bangui into three distinctive categories by classifying areas within these categories as having high, moderate, or low predicted rates of TB positivity. Among the fourteen TB clinics considered, our results identified three situated in high-risk regions, eight in medium-risk zones, and three in low-risk areas. This assessment, allowing for a stratified approach, may have the potential benefits of targeting support and resource allocation to facilities located in high-risk regions, ensuring sufficient capacity for diagnosis, treatment, and prevention and resources for moderate-risk regions as well. Additionally, the practicality of applying these results to decision making to guide research is clear. For example, it can be used to identify the sites depending on the goal of the study.

Intriguingly, both observed and predicted TB positivity rates showed a weak negative correlation with the HIV incidence rate, children underweight, stunted growth, distance to major roads, and elevation. These negative relationships were statistically significant, indicating a counterintuitive result and also reflecting the typical bias associated with notification data from passive case findings. Despite these counterintuitive correlations, the model remains useful in highlighting patterns that may reflect access-to-care inequalities rather than true transmission dynamics, thus informing targeted investigations and interventions. Increased distance from major road networks (remote location), poor nutritional status among children, and a high incidence of HIV could mean low socioeconomic status, low access to health care, and poorer health-seeking behavior. These factors may lead to fewer people actively seeking care for TB, a disease that could remain asymptomatic for long periods of time. For these reasons, such areas may give a false image of low TB transmission, while the reverse may be true for areas with relatively better socioeconomic indicators. The established association between HIV, child underweight, stunted growth, and TB highlights the need for further research to uncover the underlying mechanisms and potential confounding variables in these relationships. It also underscores the need for active case-finding and community-based interventions to test the reliability of such modeling exercises in low-resource settings.

Discrepancies observed between predicted and reported TB positivity rates at the clinic level may arise from several factors. These include data quality issues, such as under-reporting or delays in case notification, and health-seeking behavior patterns, where patients may bypass the nearest facility for reasons such as stigma, service availability, or perceived quality of care. Additionally, local epidemiological or environmental factors not captured in the model—such as informal settlements, recent displacements, or facility-level diagnostic capacity—may contribute to mismatches. While this proof-of-concept study focused on identifying spatial patterns, future phases will include field validation and deeper integration of programmatic data to better understand and address such discrepancies. Therefore, the current findings should be seen as preliminary indications to guide future investigations rather than definitive programmatic recommendations at this stage.

A notable strength of the Bayesian model was its ability to address potential limitations associated with passive case-finding data collected from individual facilities, which can be influenced by variations in diagnostic capabilities across the city. The Bayesian model addressed this issue by incorporating the local socio-demographic context into its predictions.

However, during the comparison of observed and predicted TB positivity rates, we encountered a challenge, as expressing rates on a common scale proved to be complex. To compare risk levels, our categorization approach, low, medium, and high, while pragmatic, may have introduced limitations in the granularity of our risk assessment. Additionally, it is noteworthy that the potential for variability in diagnostic criteria and practices across different facilities may still introduce some degree of remaining uncertainty into the risk categorization process. Future iterations of the model could explore finer stratifications or continuous risk surfaces to enhance decision making in densely populated or heterogeneous areas.

The Bayesian inference models displayed adaptability when challenged with disparities between quartier names in the shapefile data and recorded client addresses. These models effectively addressed these issues by providing predictive outputs for all areas, including those with missing data, and allowed us to predict TB positivity rates for each quartier, thereby ensuring comprehensive coverage in predictive outputs. However, the challenge related to neighborhood name disparities, whilst effectively addressed by our Bayesian inference models, did introduce limitations, including reduced sample size and missing data, which may, in turn, have implications for the precision of predictions in affected areas. In addition, while standard classification metrics such as accuracy, recall, and F1-score are commonly used in AI-driven disease prediction, their applicability in spatial epidemiological modeling is limited. However, their selective integration—when adapted to spatial strata—could still offer complementary insights when combined with spatially relevant performance indicators. Unlike traditional disease classification models that assign binary or categorical outcomes to individual cases, our approach predicts continuous spatial risk levels across different areas, which do not always fit neatly into standard classification frameworks. Incorporating additional performance perspectives could be beneficial for refining model validation in future studies. For instance, region-specific precision–recall analysis could help evaluate how well the model differentiates between high-risk and low-risk zones. Similarly, uncertainty quantification techniques such as Bayesian credible intervals could enhance confidence in spatial risk predictions. Future work could explore hybrid evaluation approaches that combine risk stratification metrics with more conventional AI evaluation measures while maintaining interpretability for public health applications. Given the data limitations typical of low-resource, high-burden settings such as Bangui, our modeling approach incorporates assumptions that may introduce uncertainty. The assumption of a relatively uniform baseline TB incidence across the city, which was necessary due to limited surveillance data, may not fully reflect spatial heterogeneity in transmission. While our Bayesian framework helps capture some uncertainty through posterior distributions, we acknowledge that the full extent of uncertainty—especially from non-systematic missing data—may not be fully quantifiable. Finally, the population density data derived from sources such as WorldPop may not fully reflect recent displacement or urban migration patterns in Bangui due to ongoing conflict and instability. As such, some degree of misclassification or underestimation in certain areas is possible. Despite this, our model provides indicative relative risk estimates and highlights areas where improved surveillance and field validation would be particularly beneficial to strengthen the reliability of findings.

The formal model validation is constrained by the lack of historical disaggregated TB data. We intend to assess the accuracy of our Bayesian model for CAR with a specific focus on the TB clinics that showed differences between observed classifications and predictions, comparing the number of TB clinics that change classification after evaluation. This evaluation will involve time series cross-validation to gauge the performance of AI predictions against government TB notification cases over time. Beyond this, AI applications in the domain of TB surveillance offer promising avenues for improvement. Specifically, integrating genomic data from TB strains, with a particular focus on drug resistance, would provide an opportunity to enhance the predictive capabilities of AI models dedicated to assessing and forecasting MDR-TB burdens. Notably, MDR-TB is significantly under-reported in RCA, and the availability of GeneXpert testing is still restrictive [1]. This real-time validation phase will include sensitivity analyses to explore the influence of key input variables on model predictions. Moreover, it is crucial to continuously feed the model, necessitating regular updates with programmatic or actual case data. Indeed, this ongoing nurturing process is essential for the model to evolve and become increasingly robust. These future iterations may include credible intervals to enhance uncertainty communication.

In our assessment, the inclusion of Latent Class Analysis (LCA) stands to greatly benefit our research in TB control. LCA is a statistical tool that can help identify hidden subgroups within a population, representing individuals who share common risk factors, behaviors, or vulnerabilities affecting TB transmission dynamics. By pinpointing these latent classes and evaluating their alignment with the predictions generated by our AI model, we can validate the model’s accuracy, enhance the robustness of our research findings, and provide insights into complex interactions and potential confounding factors within relationships, such as those between HIV, stunted child growth, and TB positivity.

The implementation of AI-based TB surveillance models such as the one presented in this study holds significant potential to improve public health decision making in resource-limited settings. Rather than replacing existing surveillance systems, AI should be viewed as a complementary tool that enhances the precision and responsiveness of national TB programs. However, its effectiveness remains contingent on the availability of timely and quality data; thus, strengthening routine data collection remains a prerequisite for maximizing the impact of such tools. By identifying geographic hotspots and underserved areas, the model can support targeted interventions, guide resource allocation, and inform district-level prioritization for active case finding. However, successful implementation requires a strong policy commitment to invest in data infrastructure, ensure interoperability between health information systems, and build technical capacity at the national and sub-national levels. Moreover, institutionalizing such tools within TB program operations will require close collaboration between health authorities, digital innovation partners, and policy makers. As the model is designed to be iteratively updated, it offers an opportunity for continuous learning and refinement aligned with real-time data and evolving program needs.

## 5. Conclusions

In conclusion, our study underscores the potential of high-resolution spatial analysis and predictive modeling in the context of TB control and the predicted correlation between variables and TB positivity. The ability to identify areas with high unmet needs, categorize regions by TB burden, and assess facility-specific risk may allow for a more precise and effective allocation of resources and interventions. These findings, if validated, will provide valuable insights for policymakers, healthcare practitioners, and public health officials to guide the development and implementation of tailored strategies to combat TB within Bangui and attain the NTP’s key goals by the end of 2028. Also, it would allow the scalability of the approach to other regions in the country and other lower-income settings.

## Figures and Tables

**Table 1:** Description of the variable and their source.

Variable	Description/Definition	Resolution	Source
Total population density	Number of people per square kilometer	100 m ^a^	WorldPop Hub [19]
Prevalence of underweight in Children	Percentage of children underweight (below −2 SD of weight for age according to the WHO standard)	5 × 5 km ^b^	Institute for Health Metrics and Evaluation [20]
Access to improved water source	Percentage of the de jure population living in households whose main source of drinking water is an improved source	5 × 5 km	Global Health Data Exchange [21]
Access to improved sanitation facilities	Percentage of the de jure population living in households whose main type of toilet facility is no facility (open defecation)	5 × 5 km	Global Health Data Exchange [21]
Prevalence of Stunting in Children	Percentage of children stunted (below −2 SD of height for age according to the WHO standard)	5 × 5 km	Institute for Health Metrics and Evaluation [20]
Vaccination coverage (DPT1, DPT3, and measles)	Percentage of children 12–23 months who were vaccinated	5 × 5 km	Institute for Health Metrics and Evaluation [22,23]
Literacy (men and women)	Percentage of men and women (age 15–49 years) who are literate	5 × 5 km	Global Health Data Exchange [24]
Distance to built settlements	Distance of a built settlement from the centroid of a population cluster measured in meters	100 m	WorldPop Hub [25]
Distance to major roads	Distance of a major road from the centroid of a population cluster measured in meters	100 m	WorldPop Hub [25]
Children mortality under 5	Estimates of death counts for children under-5 (0–5 years old)	5 × 5 km	Global Health Data Exchange [26]
HIV prevalence	Estimated prevalence among 15–59-year-old individuals	5 × 5 km	Global Health Data Exchange [27]
Health Facility coverage (density)	Number of health facilities per square kilometer	Point-level data	The Humanitarian Data Exchange (HDx) [28]
Night-time lights	The VIIRS data are measured in nanoWatts/cm^2^/sr	100 m	WorldPop Hub [25]
Elevation	Elevation above the sea level (in meters)	100 m	WorldPop Hub [25]

^a^ m = meters ^b^ km = kilometers.

**Table 2:** Comparison of predicted and notified TB positivity in the vicinity of TB clinics.

TB Clinic	Predicted TB Positivity	Notified TB Positivity
Petevo Centre de Santé	Medium	Low
Lakounanga Urbain Centre de Santé	High	High
Centre de Santé Saint Joseph	Low	No data available
Complexe pédiatrique	Medium	No data available
CNRISTAR CTA	Low	No data available
Castors CSU	High	High
CNHUB HN	Medium	Low
Mamadou Mbaiki Centre de Santé	High	High
Hospital Communautaire	Low	High
Obrou Fidel Camp Centre de Santé	Medium	High
Amis Afrique ONG	Medium	Low
Malimaka	Medium	Medium
Hospital Amite	Medium	Medium
Bédé Combattant CSU	Medium	High

**Table 3:** Pearson correlation coefficients computed for predicted TB.

Variable	Pearson Correlation Coefficient (with Predicted TB Positivity)	*p*-Value
Population density	0.67	<0.001
Observed TB positivity rate	0.61	<0.001
Night-time lights	0.47	<0.001
DPT 3 vaccination (%)	0.30	<0.001
DPT 1 vaccination (%)	0.30	<0.001
Children underweight (%)	−0.31	<0.001
HIV Incidence rate	−0.27	<0.001
Distance to built settlements	0.25	<0.001
Children stunted (%)	−0.23	<0.001
Measles vaccination (%)	0.21	<0.001
Access to improved water source (%)	0.205	<0.001
Lacking sanitation services (%)	0.18	<0.001
Distance to major roads	−0.13	<0.001
Elevation	−0.10	<0.001
Literacy among males (%)	0.09	<0.001
Literacy among females (%)	0.05	<0.001
Child mortality rate	0.03	<0.001

**Table 4:** Pearson correlation coefficients computed for notified TB positivity.

Variable	Pearson Correlation Coefficient (with Notified TB Positivity)	*p*-Value
Predicted TB positivity rate	0.61	<0.001
Population density	0.58	<0.001
DPT 1 vaccination (%)	0.51	<0.001
Lacking sanitation services (%)	0.43	<0.001
Children underweight (%)	−0.42	<0.001
Children stunted (%)	−0.41	<0.001
Distance to major roads	−0.37	<0.001
Night-time lights	0.32	<0.001
DPT 3 vaccination (%)	0.32	<0.001
Measles vaccination (%)	0.31	<0.001
Literacy among males (%)	0.25	<0.001
Access to improved water source (%)	0.22	<0.001
Elevation	−0.18	<0.001
HIV Incidence rate	−0.17	<0.001
Literacy among females (%)	0.12	<0.001
Child mortality rate	0.09	<0.001
Distance to built settlements	0.07	<0.001

## Data Availability

The data that support the findings of the study are available from the National Tuberculosis Programme Department of CAR upon reasonable request.
